# Drivers of vulnerability to medicine smuggling and combat strategies: a qualitative study based on online news media analysis in Iran

**DOI:** 10.1186/s12913-024-10805-7

**Published:** 2024-03-27

**Authors:** NourolHoda Fakhrzad, Vahid Yazdi-Feyzabadi, Maryam Fakhrzad

**Affiliations:** 1 Health Services Management Research Center, Institute for Futures Studies in Health, Kerman University of Medical Sciences, Kerman, Iran; 2 Social Determinants of Health Research Center, Institute for Futures Studies in Health, Kerman University of Medical Sciences, Kerman, Iran; 3 Virtual School, Center of Excellence for E-Learning, Vice-Chancellery for Education, Shiraz University of Medical Sciences, Shiraz, Iran

**Keywords:** Medicine smuggling, Drivers, Combat strategies, Media analysis, Iran

## Abstract

**Background:**

Medicine smuggling poses a serious public health threat, limiting patients’ safe and timely access to this essential resource. Thus, this study aims to identify the factors contributing to the vulnerability to medicine smuggling and propose effective strategies to combat this issue in Iran.

**Methods:**

An analysis of news media was conducted using qualitative content analysis. News text items related to medicine smuggling were retrieved from various online news sources between March 21, 2017, and May 21, 2023. To select health-oriented and general online news stations, news agencies, and newspapers, the purposeful sampling method with a maximum variation strategy was used. The selected sources included Mehr News Agency, Khabar Online, Islamic Consultative Assembly News Agency (ICANA), Islamic Republic News Agency (IRNA), Iranian Students News Agency (ISNA), Hamshahri, Donya-e-Eqtesad newspapers, Webda, Sepid Online, and Iran’s Food and Drug Administration News Agency (IFDANA). All data coding was manually done using Microsoft Excel software version 2016.

**Results:**

A total of 277 news articles were found to meet the established criteria for inclusion. The analysis revealed four main themes, each with several sub-themes, that shed light on the factors that drive vulnerability and the strategies to combat medicine smuggling. These themes are the economic environment, government and stewardship, information technology systems, and socio-cultural factors. The economic environment emerged as the most significant theme, encompassing medicine selection, reimbursement, and procurement, all of which affect the smuggling of pharmaceuticals in Iran.

**Conclusion:**

To combat medicine smuggling, it is important to adjust policies based on the identified vulnerabilities. Effective strategies to reverse pharmaceutical smuggling include capacity building of pharmaceutical manufacturing companies, implementing regulated and enhanced supervisory and rulemaking policies, strengthening health insurance, improving e-infrastructure, and increasing public awareness through collaborative approaches involving various stakeholders within and outside the health system.

**Supplementary Information:**

The online version contains supplementary material available at 10.1186/s12913-024-10805-7.

## Background

The illicit production, transportation, or sale of typically legal goods poses a significant and increasing threat to society and the global economy [1, 2]. These activities refer to a variety of criminal offenses, such as smuggling, trafficking, and counterfeiting. Smuggling involves the illegal transportation of legitimate products, while trafficking refers to the unlawful transportation of illegal items such as counterfeits [3]. The illicit global trade has serious consequences, including the growth of organized criminal activities, financing of terrorist organizations, increased corruption, and endangerment of public health and state institutions [4].

The term “black market” refers to the illegal economy where people engage in activities related to the production, importation, exportation, distribution, sale, or purchase of goods, often to earn money. This type of economy often involves moving goods across state or country lines for profit [5].

Ensuring the provision of top-notch healthcare services is not feasible in the absence of top-quality medications [6]. Access to medicine is crucial for high-quality healthcare, significantly impacting human health. Universal Health Coverage (UHC) aims to ensure that everyone can access essential healthcare services without financial barriers [7]. Access to medicine is emphasized in Sustainable Development Goal 3.8, which aims to achieve universal health coverage through “access to safe, effective, quality, and affordable essential medicines and vaccines for all” [8].

Medicine smuggling encompasses illegal importation or exportation of drugs, as well as unlawful transportation across borders. Reverse smuggling involves moving medication out of a country for non-humanitarian reasons, such as political insecurity or profit. Importing or exporting counterfeit or illegal medicines into a country’s official pharmaceutical market can have grave health consequences, including potential fatalities [9]. It may hinder the affordability and adequacy of necessary and essential medicines lead to increased unmet pharmaceutical needs and eventually cause harmful public health consequences for patients [10]. Economically, medicine smuggling imposes costs on the health system and households [11]. Pharmaceutical expenses are a major cause of out-of-pocket payments and catastrophic health expenditures, which can lead to households falling into poverty as a result of severe or chronic illnesses. As a result, this reduces people’s access to medicine [12]. Outpatient medications and supplies represent more than 30% of all out-of-pocket costs, which is about twice the cost of inpatient services [13]. These statistics show that drugs account for a significant portion of healthcare costs and patients face high out-of-pocket expenses for pharmaceuticals.

In Iran, recent reports indicate that medicine and fuel account for the largest proportion of trafficked goods estimated at $7 billion annually [9]. Several studies have highlighted the various factors responsible for the shortage of pharmaceuticals in Iran. These include political and cultural threats, economic sanctions, high inflation, the sale of medicines at multiple prices, poor management of consumer demand for medicines, restrictive laws, and the growth of smuggling networks. The literature emphasizes the need for more effective control to prevent the black market of drugs from flourishing [14–24]. Studies have shown that cultural and social factors contribute to medicine smuggling and trafficking, making good smuggling penalties ineffective as a deterrent [23, 24].

Although there are some reasons for medicine smuggling, the context-specific nature of the phenomenon means it is affected by a country’s policies and reforms [21, 25].

Media content analysis is one of the applied research methods [26–34] that can analyze the content to understand an issue and propose solutions to solve the issue. In this regard, various studies have been conducted in Iran [27, 28, 35] and the world [26, 29–33]. Based on this, media content analysis has been used in this research.

Iran, in recent years, faced economic sanctions and banking transaction hardships, which constrained trade exchange and foreign exchange reserves. Recent fluctuations in the foreign exchange rate prompted pharmaceutical subsidies and exchange reforms, shifting preferential exchange allocation from manufacturers and importers to end consumers. However, there remains a paucity of evidence regarding medicine smuggling in Iran. A study conducted through news media analysis aimed to answer two questions: (1) What drivers make Iran’s health system vulnerable to medicine smuggling? and (2) What are the combat strategies to confront medicine trafficking in Iran?

### Context

Iran, with an estimated Gross Domestic Product (GDP) of approximately $388.54 billion in 2022 and a population of around 88.5 million inhabitants, ranks as the fifth-largest economy in the Middle East and North Africa (MENA) region [36]. The Iranian healthcare system comprises three tiers: primary, secondary, and tertiary [37]. Healthcare financing sources include government allocations, general taxation, health insurance, donations, and out-of-pocket payments. Despite legislation to establish a unified national insurance scheme in 2010, the physical integration of health insurance funds was never fully implemented [38].

Iran’s current expenditure on pharmaceuticals accounts for a substantial portion, ranging from 25 to 65%, of the total healthcare spending across both public and private sectors, a pattern commonly observed in many developing countries [39, 40].

By regulations of Medical and Pharmaceutical Affairs, initially enacted in 1955 and subsequently modified, all activities related to the pharmaceutical sector, encompassing production, importation, distribution, and pharmaceutical pricing, are subject to direct oversight by Iran’s Food and Drug Administration (IFDA) [41]. Iran’s pharmaceutical policy was originally rooted in a compulsory generic pharmaceutical approach during the years following the Islamic Revolution [40, 42]. However, since 2001, there has been a shift in policy towards the promotion of competition within the domestic pharmaceutical industry and the enhancement of medication quality, resulting in the adoption of a branded generic policy [40]. A substantial portion of Iran’s pharmaceutical market is currently under the influence of semi-governmental entities, with the private sector contributing less than 50% to Iran’s pharmaceutical market share [42]. In any case, the domestic pharmaceutical industry still maintains control over approximately 60% of Iran’s overall pharmaceutical market, while the remaining 40% primarily comprises the expenses associated with high-priced, advanced imported pharmaceuticals [40]. It’s worth noting that all prescriptions and Over-The-Counter (OTC) medicines intended for Iran’s pharmaceutical market must be included in the Iran National Drug List (NDL) before their registration with addressing criteria such as quality, effectiveness, safety, and cost-effectiveness of these medications [41].

In recent times, Iran has allocated around 9% of its GDP to healthcare (equivalent to approximately US$ 475 per capita), with about 1.4% (equivalent to US$ 75 per capita) dedicated to the pharmaceutical sector [43, 44]. In 2018, there were 185 Iranian pharmaceutical manufacturing companies, 232 importers, and 50 distributors [45]. While the proportion of locally manufactured pharmaceuticals in Iran exceeds 95% in terms of sales volume [45, 46], the domestic manufacturers’ share of the pharmaceutical market value remains relatively low due to reliance on imported raw materials for production. Iran has a pharmaceutical price regulation system utilizing reference and cost-plus pricing systems. Different markups at the pharmacy, distributor, and importer levels are used with lower markups for high-priced drugs [47].

A substantial portion of the active pharmaceutical ingredients (API) is produced within the country, with the remainder sourced from reputable companies in India, China, and occasionally European companies [40]. While previous statistics indicated that domestically produced drugs constituted over 70% of Iran’s pharmaceutical market value, a recent report by the Research Center of the Islamic Parliament cited a figure of 23% [48]. For foreign currency transactions, a minimum of three distinct exchange rates are utilized as reference points. The first rate is the official one, which remains fixed at 42,000 Iranian rials per 1 US dollar and is primarily designed to facilitate the importation of essential goods. The second rate is the Forex Management Integrated System (NIMA) rate, which can be considered a quasi-market rate. The management of the NIMA rate falls under the jurisdiction of the Central Bank of Iran (CBI), and it mandates major exporters, including those in the petrochemical sector, to route their export earnings through this system. Finally, other minor foreign currency requirements, such as those related to travel and cash transactions, are fulfilled through a parallel market facilitated by banks and currency exchange establishments, albeit for limited amounts [49].

## Method

### Study design

For this qualitative study, we utilized the content analysis approach, which includes media content analysis. Our objective was to investigate the factors driving medicine trafficking in the Islamic Republic of Iran and explore the strategies to combat it. We analyzed the content of online news media to answer these questions.

### Sources of data and inclusion criteria

To collect data, we analyzed various news media sources, including specific online news agencies such as Mehr News Agency, Khabar Online, ICANA, IRNA, and ISNA. We also examined two newspapers that focus on social and economic issues, namely Hamshahri and Donya-e-Eqtesad, as well as three sources that cover health news and events, namely Webda, Sepid Online, and IFDANA. To select the news sources, we used a purposeful sampling method with a maximum variation strategy. We searched for relevant headlines from March 21, 2017, to May 21, 2023, which spans six years of the Solar Hijri calendar. We took into account the several periods of exchange rate volatility in Iran since approximately 2016-17 when the exchange rate varied from about 41,000 Rials to 520,000 Rials per US dollar, which has had consequences for the economy and health.

The study utilized a keyword search strategy to identify relevant content for analysis. The keywords used included “medicine”, “pharmaceuticals”, “smuggling”, “trafficking,” “black market,” and “medicine smuggling/trafficking” in Persian. As some of the news sites have no advanced and reliable search engine, we inevitably used single-keyword to retrieve the news text items to ensure that no relevant headlines were missed.

#### Inclusion criteria

Criteria for entering the news included:

- News items were textual and subject to factors affecting and driving the medicine smuggling and its combat strategies.
- News reported in the Persian language and within the period of March 21, 2017, to May 21, 2023.

#### Exclusion criteria

Criteria for excluding news were as follows:

- News videos, voices, and multimedia platforms.
- News of pharmaceutical exports or imports that were not subject to medicine smuggling.
- news related to cosmetics, narcotics, and addictive substances or medicines that were not subject to therapeutic use,
- news that was about the detection and destruction of smuggled medicines and was not subject to the aim of the study.
- News that was about medicine smuggling and counterfeit medicines, but did not clearly explain challenges and solutions.

**Fig. 1:**
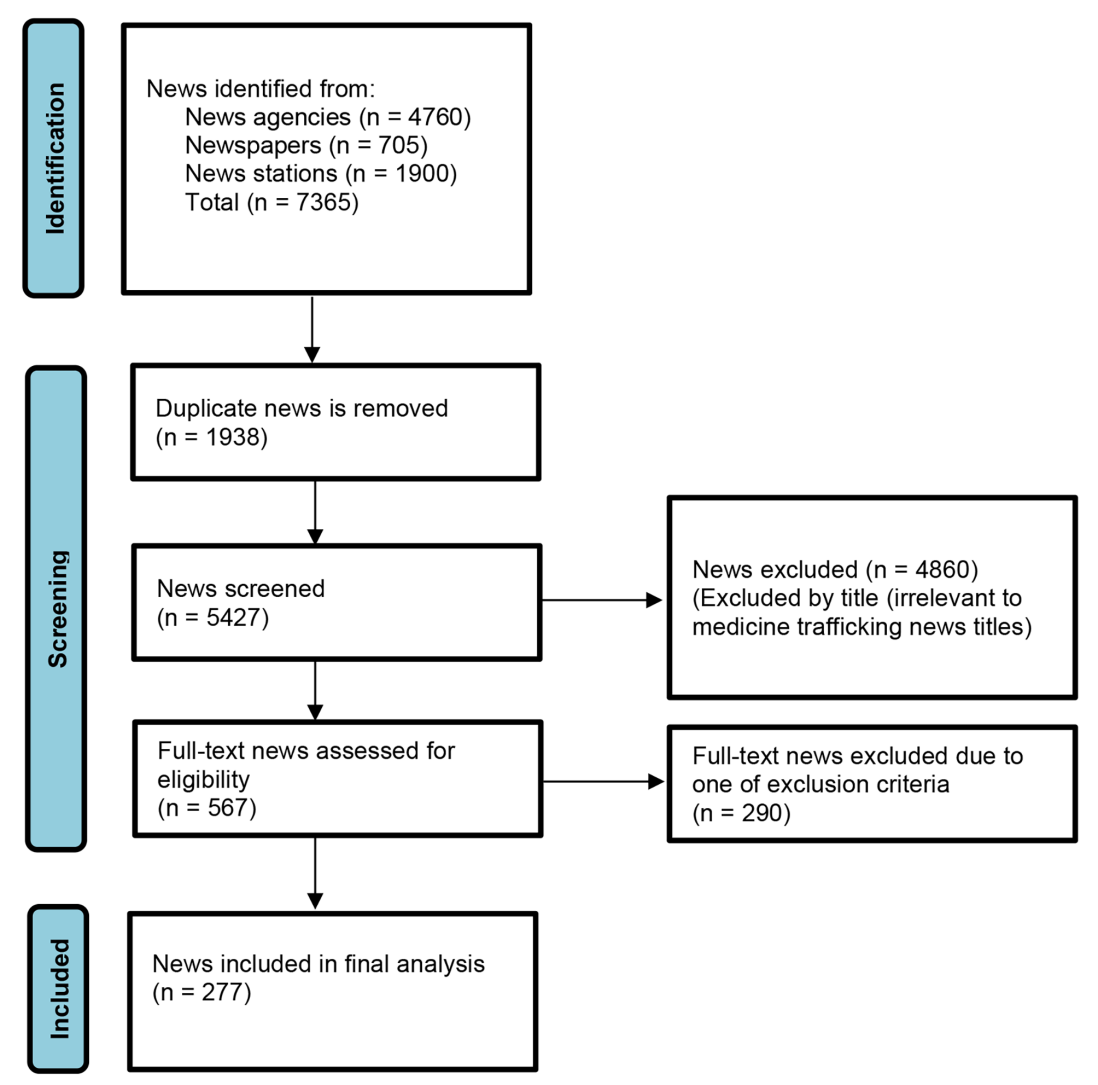
PRISMA diagram for the news screening

To ensure that only relevant content was included in the analysis, a rigorous search strategy was employed. A total of 7365 textual news items were collected from various news stations, news agencies, and newspapers. After removing duplicates (*n* = 1938), 5427 unique news items remained. The preliminary screening was conducted by the first and second authors based on the titles of all retrieved news text items. A total of 567 news items met the inclusion criteria based on their titles and were retained. The full text of these news items was downloaded and assessed for relevance. Finally, after excluding irrelevant news related to the subject, 277 news items were included in the final analysis (Fig. 1).

### Data extraction and analysis

To ease collaboration during the screening and data extraction stages, all remaining news items were transferred to a single sheet. The corresponding author, along with the first and third authors, extracted data from included news items using a uniform data extraction sheet within Microsoft Excel software 2013 (Table S1). The news item characteristics included the news title, source, time of publication, name of news media, interviewee or newscaster, the full text of the news, and emerged codes. The data were analyzed using thematic content analysis with an inductive data-driven approach, following the procedure outlined by Braun and Clarke. The procedure involves a continuous analysis and review between data and encodings, performed during analyses. The process consisted of six steps, which are as follows:

1. Familiarizing with the data: In this step, the news texts were carefully read and reviewed to understand the information provided.
2. Creating the initial codes: After reviewing the news, the key points related to the challenges and strategies of medicine smuggling were identified and categorized.
3. Searching for selective codes: Overlapping codes were summarized into a code set.
4. Forming sub-themes: These codes were then categorized densely and placed under the sub-category title.
5. Defining and naming the main themes: Sub-classes were evaluated and grouped under the four main themes: economic environment, governance and stewardship, information technology systems, and socio-cultural factors.
6. Finally, the report was prepared [50]. All the relevant news text items were manually coded.

#### Trustworthiness

Four criteria proposed by Guba and Lincolns [51], including credibility, transferability, dependability, and confirmability were used to trustworthiness the results. We employed several methods to ensure the credibility of our study. Member checking, negative case analysis, and referential adequacy were used to verify the accuracy of our findings. To ensure dependability, we created detailed drafts of the study protocol throughout the research process and measured the coding accuracy and inter-coders’ reliability of the research team. We ensured confirmability by holding weekly investigators’ meetings and using triangulation techniques, both theoretical and investigative, to cross-check our findings. We also transparently described the research steps taken from the beginning of the project to the development and reporting of the findings. To increase the transferability of our results, we quantified data saturation.

## Results

**Fig. 2:**
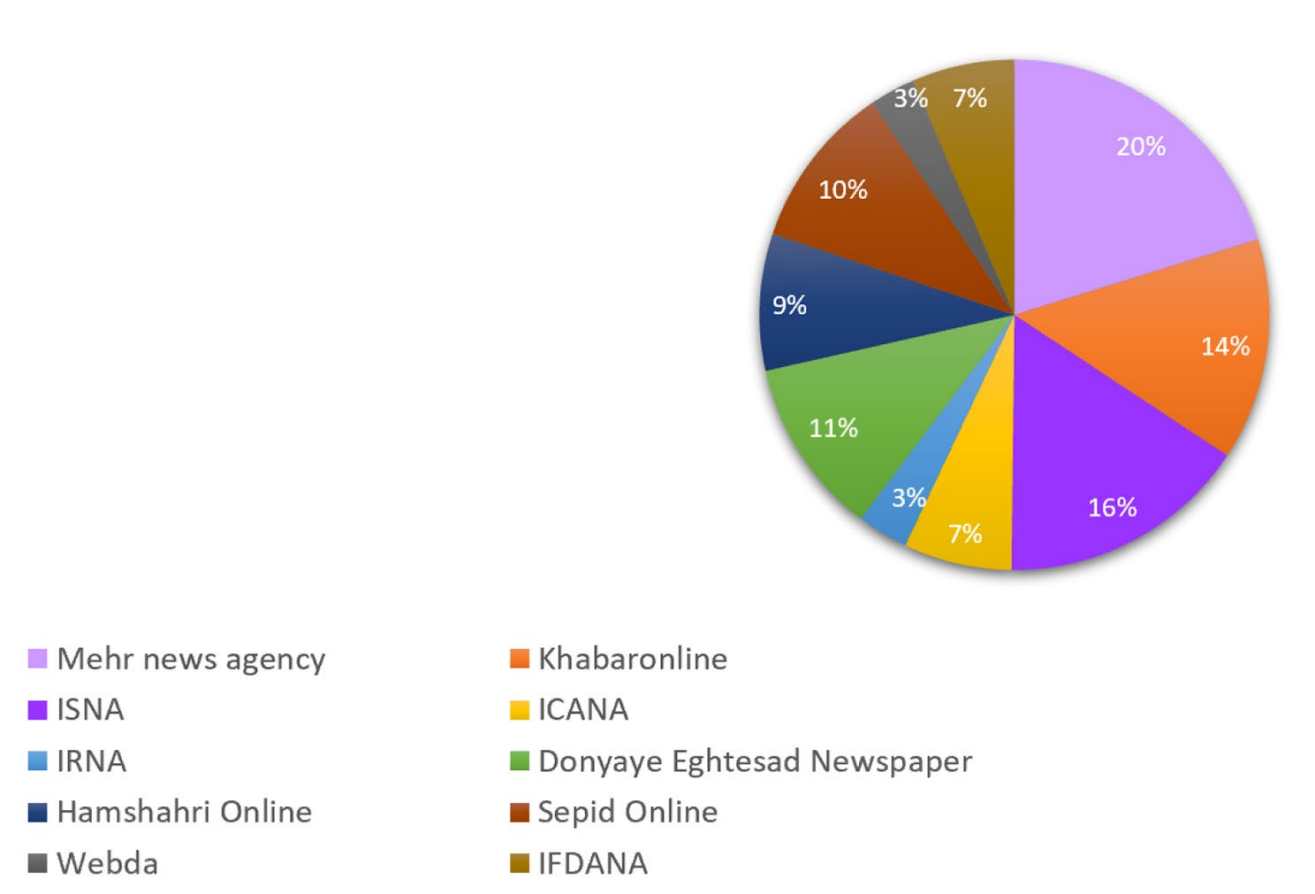
Proportion (%) of the analyzed news items about medicine smuggling in Iran by news sources

After reviewing 10 news sites, official news agencies, and related newspapers, it was found that there were a total of 277 reports on medicine smuggling. The frequency of these reports is shown as a percentage in Fig. 2. The figure highlights that Mehr News Agency and ISNA had the highest number of news headlines related to medicine smuggling, whereas Webda and IRNA had the lowest.

**Fig. 3:**
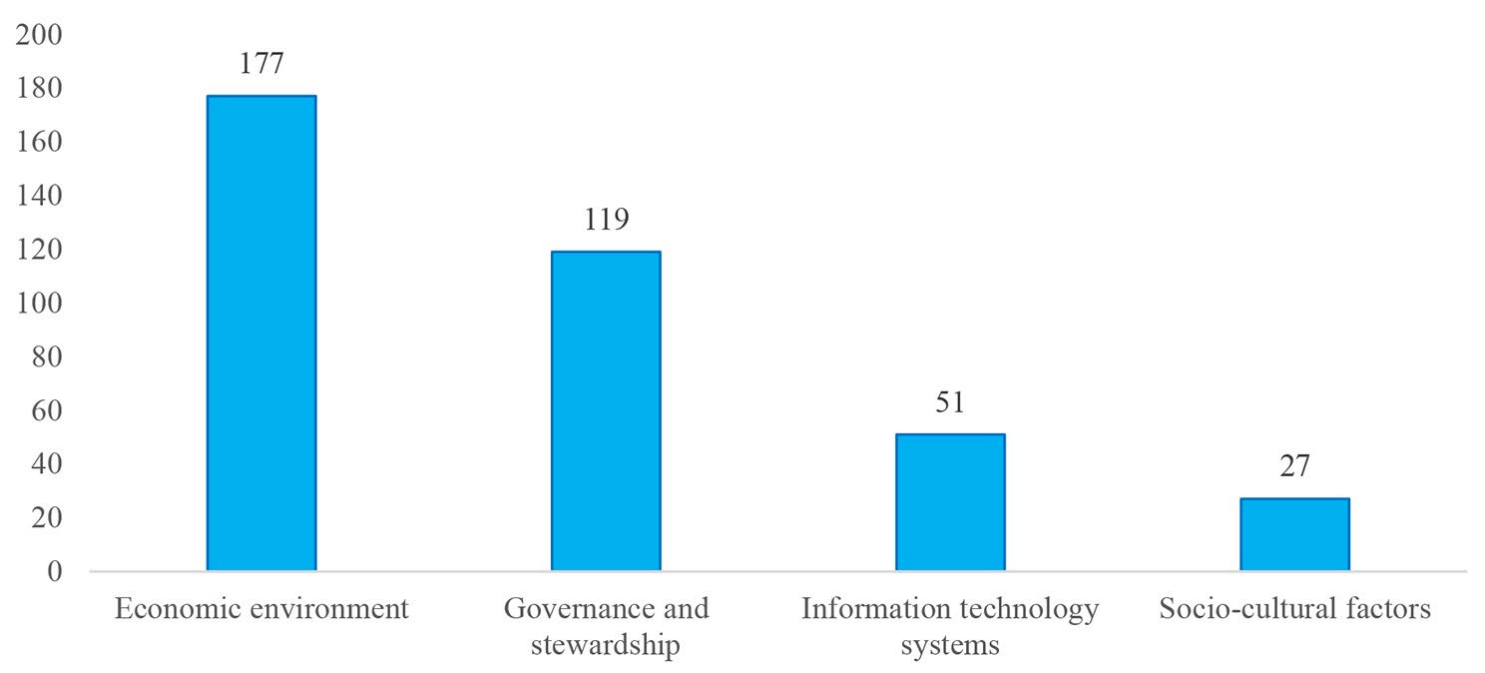
Frequency of the codes related to each emerged theme for drivers of vulnerability and combat strategies to medicine smuggling

According to Fig. 3, the economic environment received the most press coverage among news headlines, while socio-cultural factors received the least amount of coverage.

The findings regarding drivers and combat strategies were grouped into four main themes and seven sub-themes, as shown in Table 1. The four themes are the economic environment, governance and stewardship, information technology systems, and socio-cultural factors.

**Table 1:** Emerged themes, subthemes, and codes related to drivers of vulnerability to medicine smuggling in Iran

Theme	Sub-theme	Drivers of vulnerability to smuggling	Combat strategies
**Economic environment**	Medicine selection and reimbursement	• Unrealistic prices of locally manufactured medicines • Defective pharmaceutical pricing mechanism • Multiple exchange rates • Financial incentives of the pharmaceutical market • Liquidity problem of the pharmaceutical industry • Insurers’ debt to pharmacies	• Pricing mechanisms reform • Strengthening and capacity building of health insurance • Exchange policies reform • Reforming pharmaceutical exchange policies
	Medicine procurement	• Increased black market • Imposition of economic sanctions	• Financial and knowledge-oriented support of the pharmaceutical industry • Improving the banking system
**Governance and stewardship**	Targeted supervision and control	• Weak intersectoral collaboration • Low authority of the Ministry of Health • Black-market distributors • Physicians’ preference to prescribe brand medications • No adherence to clinical guidelines • Increased induced demand • Poor border control • Collusion and bribery by executive staff/officer	• Strengthening control of borders • Strengthening inter-sectoral/ agency collaboration • Improved intelligent and digital monitoring systems • Developed and institutionalized administrative health audit initiative
	Directive strategies and policies	• Shortcomings in the pharmaceutical supply and distribution chain • Insufficient supervisory manpower • Inefficient pharmaceutical subsidy policy	• Imposition of duties or tariffs for the subsidized goods • Outsourcing of supervision of pharmacies
	Legislation and regulation	• Outdated and old-fashioned anti-trafficking/smuggling laws • Conflict of interests in the pharmaceutical system • Low political will	• Inquiring about the authenticity of pharmaceutical products • Updated and deterrent laws and penalties • Prohibition of prescribing medicines out of the Pharmacopoeia list • Identification of health-oriented products with a unique code
**Information technology systems**	Electronic pharmaceutical information systems	• Shortcomings of the electronic prescription scheme • Flaws in TTAC* and its security vulnerabilities • Lack of electronic infrastructure in the pharmacy • Lack of linkage with the electronic information systems of other organizations • Lack of linkage between the TTAC system and electronic prescription systems	• Electronic prescription systems • Using the capacity of electronic health records • To link between fragmented pharmaceutical information systems • Fixing of software and system bugs
**Socio-cultural factors**	Awareness raising of patients and providers	• Poor cultural persuasion of patients and providers • Poor dissemination of accurate/clear information • Irrational medication use • Poor public awareness about smuggling	• Public awareness about the consequences of illicit medicine smuggling • Public awareness about the TTAC system • Eliminating the motivation for smuggling by destroying discovered smuggled medicines

* TTAC: Tracing, Tracking, and Authentication Control.

### Theme 1: economic environment

The pharmaceutical sector is highly reliant on a variety of economic factors within and outside the health system. These factors ultimately determine the cost of pharmaceuticals due to the high-tech, highly professional, and financially interesting nature of the industry. The various stages involved in the medicine chain such as research and development of new drugs, clinical trials, patent filing, manufacture, registration, selection of essential medicines, procurement, and distribution, inspection of manufacturers and distributors, prescribing, dispensing, pharmacovigilance, and medicine promotion control are all affected by these economic factors. The two major categories of these economic factors are medicine selection and reimbursement, and medicine procurement.

### 1–1 Medicine selection and reimbursement

Selecting essential medicines that cater to the healthcare needs of the majority of the population, determining the prices of pharmaceutical products, and arranging for their reimbursement are the key aspects of the medicine selection and reimbursement process. This intricate process involves multiple stakeholders, such as pharmaceutical companies, insurers, government bodies, the Iranian Food and Drug Administration (IFDA), and patients.

The susceptibility to medicine smuggling may be attributed to an imperfect and insufficient pricing system. Recent reports suggest that Iran’s pharmaceutical pricing system is prescriptive. It relies on reference pricing and negotiation for imported pharmaceuticals (IPs) while using a cost-plus method for domestically produced medicines. Several issues contribute to this problem, including a lack of transparency, non-compliance with regulations, a weak framework to prevent conflicts of interest, and dependence on the expertise of the members of the Pharmaceutical Pricing Commission within the IFDA. These factors may lead to the establishment of unrealistic prices.

*“Even when the increase of medicine gets into the agenda, the process often takes so much time that severely harms the industry. Moreover, the cost of competition in these pricing mechanisms has not been observed. For this reason, pricing should not be mandated and prescriptive and should be market-based.” (Mehr News Agency)*.

Iran’s economy has been hit with various economic sanctions which have caused restrictions on foreign exchange transactions and money transfers. As a result, the pharmaceutical sector has also been affected, leading the government to allocate foreign exchange to importers and producers in the pharmaceutical industry at a lower preferential exchange rate of 4,200 Iranian rials to improve people’s financial and economic access to essential goods such as medicine. However, the existence of multiple exchange rates for medicines and medical equipment has turned into a factor inflaming market volatility and corruption rather than facilitating the pharmaceutical industry. This has led to an increase in reverse smuggling where medicines, purchased with the government’s subsidized exchange rate, are sold to other countries at a higher price, instead of being distributed in the domestic market. The stakeholders in the pharmaceutical sector believe that the government’s policy of multiple exchange rates serves both as a tool for managing a country’s balance of payments and as a mechanism for providing subsidies and imposing taxes. However, this policy has exacerbated corruption within the pharmaceutical and medical equipment supply chain.

In Iran, the exchange rate difference between the free market and the government’s preferential exchange rate in the years 2019 and 2020 resulted in various challenges within the pharmaceutical sector, including delays in obtaining government-provided foreign exchange by the central bank, consequently reverse smuggling, medicine shortages, and rising medicine prices.

One solution to combat corruption and economic rent-seeking in the foreign exchange sector is the implementation of the “DAROOYAR” plan or pharmaceutical exchange/subsidy reform policy by the government, initiated in August 2022. In this plan, preferential foreign exchange is replaced with a NIMA exchange rate with a higher value, and the value difference was allocated to basic health insurance companies in rial value aiming for no increase of pharmaceutical out-of-pocket payments. In other words, the subsidy allocated was transferred from the beginning of the pharmaceutical supply chain (i.e., importers, and manufacturers) to the end of the supply chain (i.e., patients as the ultimate consumers). As a result, the policy aims to eliminate corruption, rent-seeking, and reverse drug smuggling, which arises from the remarkable value difference in the exchange rate between the free market and the government exchange rate. Due to the reform of government subsidies, locally produced medicines became subject to price increases, and these price adjustments were compensated by basic health insurance.

*“By reforming the exchange rate policies of pharmaceuticals through the Darooyar plan, we seek to prevent the impact of price increases on patients. The plan involves allocating the medicine subsidy to insurance companies, allowing organizations such as the Planning and Budget Organization to pay basic health insurance companies and then pharmacies, and this change in medicine prices will not affect patients directly.” (Mehr News Agency)*.

Utilizing the capacity of basic health insurers is one of the strategies outlined in the DAROOYAR plan, as revealed in news reports.

*“The subsidy for locally produced medicine is provided in the Rial currency, not foreign exchange (US dollar). This subsidy is allocated to insurance companies instead of imports and manufacturers. Because everyone is insured, pharmaceutical out-of-pocket payments do not increase for consumers, and in some cases, they may even be lower.” (ISNA)*.

There is a liquidity issue in the pharmaceutical industry, which prevents manufacturers from producing drugs, contributing to unrealistic pricing.

*“One of the pressing issues in the pharmaceutical industry of Iran is the liquidity problem. Pharmaceutical companies dependent on domestic sales have faced a liquidity crisis in covering production costs, primarily due to the unrealistic prices of domestically produced medicine. In other words, domestically produced drugs are so affordable that they have led to the smuggling of these drugs across geographical borders.” (ISNA)*.

Extensive financial transactions and exchange rate incentives contribute to medicine smuggling in the pharmaceutical market, as reported in the news.

*“Today, the dark shadow over the country’s pharmaceutical industry is the presence of individuals engaging in medicine smuggling, both into and out of the country. They engage in extensive financial transactions, which have allowed the black market, known as* Nasser Khosrow,*’ Street to continue operating vigorously for several years. Whenever a patient despairs of obtaining their necessary medication through official channels, they can easily procure it by visiting the Naser Khosrow market.” (Mehr News Agency)*.

The news has been highlighting a serious issue where insurance companies are failing to reimburse pharmacies. As a result, pharmacies are losing their purchasing power for medications and patients are being forced to resort to illegal markets to obtain essential medicines.

*“Pharmacies lack the purchasing power for specific medications due to insurance debt and economic exhaustion.” (Sepid Online)”*.

### 1-2- medicine procurement

The process of obtaining resources through purchase from manufacturers, suppliers, or distributors is known as medicine procurement. In many countries, pharmaceutical expenses make up a significant portion of healthcare costs. Therefore, it is essential to ensure effective medicine procurement to maintain access to medicines and control total healthcare expenditure. However, economic sanctions and black markets in the pharmaceutical sector can pose significant challenges to medicine procurement, making it more susceptible to medicine smuggling.

*“The imposition of sanctions has created obstacles for the swift repatriation of revenue generated from medicine sales, thereby presenting difficulties for domestic pharmaceutical companies. Consequently, this circumstance has contributed to a surge in the illicit exportation of domestically manufactured medications, leading to an increase in the smuggling of Iranian drugs across international borders. (Mehr News Agency)*

*”Rent, corruption, and monopolies create a black market for medicine, where counterfeit drugs are hoarded in hidden warehouses and distributed to the people” (Khabaronline).*

To enhance equitable medicine procurement, it is essential to provide financial support and strengthen the research and development base of local pharmaceutical manufacturers. By doing so, we can reduce our reliance on medicine imports and promote local production standards. This would mitigate the issue of foreign exchange allocation and address medicine shortages in many cases. Moreover, it would lead to a decrease in demand for illegal medicine markets.

*“We are committed to supporting domestic production in the pharmaceutical sector.… Instead of importing drugs at ten times the price, we should strive, with serious assistance from knowledge-based companies, to provide the same quality medication to the people at one-tenth the price.” (Donya-e-Eghtesad Newspaper)*.

Another solution in the face of sanctions is improving Iran’s banking system for fund transfers during economic sanctions, as reported in the news.

*“With the onset of economic sanctions, the issue of financial transactions is seriously discussed, especially for domestic manufacturers that lack a specific mechanism for generating funds.… Import companies have good mechanisms for fund transfers, but domestic industries will undoubtedly face difficulties. Therefore, the improving banking system can be a solution for them.” (Hamshahri Online)*.

*These strategies aim to address the challenges related to medicine procurement, ensuring access to essential medications, and reducing reliance on illegal markets during sanctions.*

### Theme 2: governance and stewardship

The theme relates to the responsible and precise management of the well-being and welfare of society. The pharmaceutical sector stewardship performs three main functions, namely policy formulation, regulation mechanisms, and supervisory mechanisms. This theme comprises three sub-themes, including directive strategies and policies, legislation and regulation, and targeted supervision and control.

### 2-1- directive strategies and policies

There has been recent media coverage on the issues with the pharmaceutical supply chain, specifically on the illegal diversion of medications from official distribution channels and reverse medicine smuggling through Iran’s borders to neighboring or targeted countries. Several news reports have brought attention to this matter.

*“A significant portion of the increase in medicine consumption is related to the leakage of medicine from the official distribution channels and the reverse smuggling of medicines to other countries.” (Hamshahri Online)*.

Moreover, there is insufficient manpower within the Ministry of Health and Medical Education and IFDA to oversee the medicine smuggling. Regarding this issue, a news report mentioned:

*“Unfortunately, the Ministry of Health and Medical Education and IFDA is largely understaffed. That is, its workforce is not proportionate to the scale of drug smuggling.” (Khabaronline)*.

To combat medicine smuggling, a multi-faceted approach is necessary. One effective strategy could be the imposition of tariffs or duties on subsidized goods by agencies such as the Trade Promotion Organization of Iran (TPOI). Another factor that contributes to medicine trafficking is inefficient medicine policy. To curb this, the government should allocate foreign exchange to essential goods and services, such as pharmaceuticals, and prohibit or restrict their export to neighboring countries. Implementing tariffs or duties on these goods can prevent drug trafficking and promote legal export. It is the responsibility of relevant organizations to take these measures seriously to prevent medicine smuggling.

*“The Trade Development Organization of Iran (TPOI), in collaboration with other relevant agencies, should impose duties or tariffs on subsidized goods so that we no longer witness some profiteering.” (Khabaronline).*

*“By allocating subsidies to the medicines needed by the people, the price of medicine will decrease, and the traffickers will also take advantage of this issue and take the medicine out of Iran with their tricks” (Mehr News Agency).*

Furthermore, outsourcing the supervision of pharmacies is another solution to combat the phenomenon.

*“We have informed the Ministry of Health and Medical Education that some actions, such as the supervision of pharmacies, should be outsourced because the owner of the pharmacy can better identify who is involved in smuggling or counterfeiting.” (Sapid Newspaper)*.

These policy and strategy recommendations aim to address the challenges in the pharmaceutical supply chain, curb medicine smuggling, and enhance oversight in the healthcare sector.

### 2-2- legislation and regulation

This section discusses the laws and regulations that govern the pharmaceutical market and aims to correct market failures while achieving non-commercial goals such as promoting health and justice. Several news reports have pointed out that outdated laws against smuggling and trafficking have hurt drug trafficking issues.

*“The old-fashioned laws have facilitated pharmaceutical trafficking. These laws were effective according to the requirements of that time; however, due to changes in the monetary values, pecuniary and discretionary punishments and penalties against pharmaceutical trafficking have practically lost their effectiveness and their deterrence. Therefore, actions must be taken to revise these laws.” (Mehr News Agency)*.

Another media news declared that:

*“The laws against medicine trafficking were enacted 50 years ago and contain over 150 articles. Given that the existing anti-trafficking law lacks the required deterrence, the government should consider reviewing and submitting a bill to Parliament. This defect has led to the abuse of many traffickers.” (ICANA)*.

Conflict of interest are prevalent in Iran’s pharmaceutical sector, indicating weaknesses in regulatory functions. These conflicts exist among key players such as physicians, pharmacies, manufacturers, regulatory bodies like IFDA, and the Ministry of Health and Medical Education. Some physicians, in collaboration with pharmacies and informal markets, may have interests that conflict with regulatory authorities and the Ministry of Health’s objectives. Additionally, monopolistic pharmaceutical manufacturers’ interests may conflict with patients as the primary beneficiaries and end-users.

*“Some physicians encourage patients to acquire brand or foreign drugs. Customized and expensive prescriptions issued by certain physicians, as well as patient referrals to specific pharmacies connected to the financial interests of physicians and pharmacies, are prevalent.” (Sapid Newspaper)*.

Another issue is the low political will to combat medicine smuggling. In this regard, media news declared that:

*“While numerous warnings have been issued to authorities in this regard, there is little political will to control it. Pharmaceutical smuggling occurs across borders, and even the Anti-Smuggling Task Force lacks adequate supervision in this area.” (Mehr News Agency)*.

Proposed solutions from the news: product authentication, health-centric identification, electronic prescription, and banning OTCs. A multi-faceted approach is advocated.

*“If the product authenticity control plan is implemented, no smuggled product can enter our distribution system. Furthermore, if it does enter, it will be detectable.” (Mehr News Agency)*.

*“One of the main reasons why people resort to smuggling medicines is due to doctors prescribing foreign medicines that are not included in the country’s official medicine list. To address this issue, it is recommended to enforce a plan that prohibits the prescription of medicines outside the approved list within the country.” (Mehr News Agency)*.

*“There are numerous benefits to having goods with identification. Imagine if every health-oriented product had an identity card. By doing so, we would know the origin and consumption of these products, which would allow for more precise planning.” (ISNA)*.

These legal and regulatory changes aim to address deficiencies in the existing legal framework, enhance the effectiveness of anti-smuggling laws, mitigate conflicts of interest, and strengthen regulatory functions to promote health and justice in the pharmaceutical market.

### 2-3- targeted supervision and control

Smuggling is a complex issue that occurs both in and out of a country. In Iran, the government’s allocation of funds to drugs and related necessities makes drugs affordable, leading to the exportation of inexpensive drugs beyond the country’s borders. On the other hand, some individuals purchase expensive drugs with government currency and smuggle them out of the country for profit. The pharmaceutical sector faces challenges in terms of fluctuating exchange rates, weak border control, and lack of cooperation between relevant sectors. These challenges make it difficult to supervise and regulate the import and export of drugs effectively.

*“In free trade zones, the highest rates of drug smuggling exist, and due to weak oversight, corruption, and substantial rents, these problems have intensified… Smuggling, whether of drugs or non-drugs, does not occur unless there are loopholes in a country’s administrative, financial, and border laws.” (Mehr News Agency).*

Drug smuggling in the country is facilitated by customs officials who engage in collusion and bribery. A news report highlighted this issue.

*“Unfortunately, individuals stationed at customs sometimes turn a blind eye to collusion and the acceptance of money from smugglers. Although customs has cut off communication between employees and importers and exporters, there are still openings for the entry of smuggled goods.” (ICANA).*

*“We are now witnessing certain individuals who are the gateway for smuggling into the country through customs, and we must ask whether smugglers also have personnel within customs?” (Mehr News Agency).*

Physicians’ insufficient attention to clinical guidelines is a major issue. According to Article 28, Paragraph 1 of the Law of the Medical Council of the Islamic Republic of Iran, physicians who prescribe drugs that are not listed in the country’s drug list are subject to penalties. When physicians ignore the laws and clinical guidelines, and prescribe medications that are not available in any pharmacy, it creates a sense of security for them. They commit offenses and contribute to the induced demand in the pharmaceutical market. Patients are referred to illegal markets, which further fuels the phenomenon of smuggling. This is a serious concern that needs to be addressed.

*“Demand for drugs occurs in the prescription sector. If clinical guidelines are taken seriously and those who do not comply are dealt with, we can control the demand sector and be successful in controlling induced demands.” (Online News).*

*“Naser Khosrow drugs are supplied for induced and illegal needs. Another group of drugs available in the black market are brands that are either scarce or nonexistent in the Iranian market but are recognized worldwide, and physicians prescribe them.” (Mehr News Agency).*

Another issue within the realm of supervision is profiteering in some pharmacies, where medications purchased with government currency are diverted to the black market through intermediaries and sold at higher prices.

*“Some pharmacies had purchased a significant quantity of medication and later exported them out of the country through intermediaries. Some medications never enter the pharmacy system and are exported from the country through other mechanisms, such as cases known as ‘suitcase drug sales’.” (Donya-e-Eghtesad Newspaper).*

Another pertinent issue in the realm of supervision is the limited authority of the Ministry of Health to combat drug smuggling. News reports have highlighted this concern:

*“The Ministry of Health has no authority outside the ‘pharmacy’ to monitor and deal with drug violations, and the law does not allow the Ministry of Health to intervene.” (ISNA).*

There are several ways to address the issue of drug smuggling. One approach is to improve border control and government ownership. Another is to encourage cooperation between different departments, while also increasing oversight of drug distribution. Additionally, an administrative health assessment model could be developed to identify weak points in the issuance of legal permits for health-related products. These strategies have been suggested in news reports as potential solutions for controlling drug smuggling.

*“To combat medicine trafficking, there should be three groups assigned to monitor the issue. Firstly, the Food and Drug Organization should label each drug with a barcode that indicates whether it is genuine or smuggled, and this should be monitored closely. Secondly, the country’s borders should be subject to necessary and comprehensive control. Lastly, there should be sufficient supervision in the area of medicine distribution, particularly in pharmacies…. Preventing this phenomenon requires multidimensional cooperation among the police, insurance organizations, punitive agencies, and the Food and Drug Administration.” (Khabar Online).*

*“Identifying administrative corruption hotspots and vulnerable points in the issuance of legal permits for health-related products, designing an administrative health assessment model by competent authorities in the field of health, and pursuing necessary plans to identify vulnerable points in the executive processes of permit issuance in the food and drug sector and controlling hotspots to increase their imports are among the measures being pursued by the Food and Drug Administration.” (Mehr News Agency).*

### Theme 3: information technology systems

The theme of this text is related to electronic systems and their infrastructure. The analysis of news media shows that electronic pharmaceutical information systems provide access to safe, valid, and reliable data. This data helps to improve decision-making procedures regarding pharmaceutical needs, stocks, consumption patterns, cost coverage, and quality of production. Such systems are essential for the efficient management of the pharmaceutical supply chain.

### 3-1- electronic pharmaceutical information systems

Electronic pharmaceutical information systems are computerized processes used in the pharmaceutical industry to manage and control various aspects of medicine import, production, distribution, dispensing, and sales. One of the main reasons why the pharmaceutical sector is susceptible to medicine smuggling is the lack of an integrated information system to track pharmaceutical products. To address this issue, the Tracing, Tracking, and Authentication Control (TTAC) system was developed and established by the IFDA as a central database for tracking and tracing health-oriented goods and their foreign exchange allocation in the pharmaceutical supply chain. However, the TTAC system is incomplete and needs to be upgraded. The recent news has highlighted software bugs and flaws in TTAC, as well as security vulnerabilities and the lack of linkage with the electronic information systems of other organizations. These issues have raised questions about the purpose of designing this system and its potential vulnerability to medicine smuggling.

*“The reason for the corruptive nature of the TTAC system is the possibility of manipulation and entering desired information. In a way, even those who are not entitled and qualified to receive the foreign currency, for the import of prohibited medicines have succeeded in receiving it” (Sepid Online).*

Upgrading and revising software flaws of TTAC is an important solution proposed in the field of electronic pharmaceutical information systems.

*“By completing the TTAC and through the mechanisms considered in it, violations in the field of medicines, equipment, and medical supplies will be reduced.” (Mehr News Agency).*

The development of an “electronic prescription” system has made it possible for physicians to electronically order medicines for their patients using an electronic signature. This system sends the prescription to pharmacies, laboratories, and other diagnostic centers electronically. This new system has the potential to prevent medicine smuggling and diversion. However, experts point out that the lack of data linkage between the TTAC system and the electronic prescription system may be a challenge. They believe that electronic prescriptions are most useful when they become an integral part of the electronic health record and prescription of patients, establishing a structural and data linkage to the TTAC system. This linkage would make it possible to track the production, sale, and consumption of medications. If a medicine diverts towards smuggling, its identification becomes feasible. Unfortunately, this linkage has not been implemented yet in terms of information system architecture.

*“Merely relying on electronic prescription might not only fail to effectively control medicine smuggling but could potentially facilitate abuse by providing unauthorized access to patient’s personal information. Consequently, prescription of medications without patients’ awareness might ensue.” (Mehr News Agency)*.

Another news media highlighted:

*“Currently, pharmacies lack the necessary infrastructure for using electronic health records. In Iran, online infrastructures are not well-established, and technical issues may arise” (Online News).*

Experts believe that a comprehensive electronic health record is necessary to fully utilize the capabilities required for managing all the different steps of the medicine chain to prevent medicine smuggling. A recent news article highlighted this issue.

*“The solution to addressing the issue of medicines diversion along the official distribution chain is the development of electronic health through establishing electronic health records for patients. Currently, only patients’ prescriptions are written electronically in the system, and the medical records of patients, including their medical history and medicine use history, are not active” (Sepid Online)*.

As per the resolution passed by the parliament and the decision made by the government, the exchange of information between entities involved in international and domestic trade must be done through the Comprehensive Trade System. However, some organizations like customs continue to exchange information with various trade-related organizations such as the Ministry of Health through fragmented systems. To address this issue, a proposed solution is to centralize the system for the exchange of trade-related information across different levels.

*“The complete communication between the TTAC system and the Comprehensive Trade System, and the discontinuation of direct communication of this system with the customs system, should be implemented as soon as possible from a legal perspective” (Sapid Newspaper).*

### Theme 4: socio-cultural factors

#### 4-1- awareness raising of patients and providers

Patients and healthcare providers must have sufficient awareness about the pharmaceutical industry and its products. Poor public knowledge regarding the risks of accessing medicines from informal markets, lack of information about methods to regulate the medicines available in the country, and even inadequate public education can result in increased demand for black markets and the rise of medicine smuggling.

*“If we actively introduce the phone-based pharmaceutical information system (1490 or 190) to the people effectively and inform citizens that, in the shortest time, they can inform all the places where the required medication is available. This will decrease the referrals to the unauthorized medicine market.” (Mehr News Agency)*.

A key factor contributing to the emergence of illegal or black pharmaceutical markets is the preference of patients and physicians for branded or foreign medications over generic drugs. This issue has been widely reported in the news.

*“People sometimes support the activities of the contraband medicine market to the extent that it has been observed that a patient who is adamant about using a certain medication, for whatever reason, even if the healthcare authorities deem the drug not suitable for prescription, goes to the Naser Khosrow black market to obtain it. This is while the medicine may not be suitable for supply according to health authorities.” (ICANA)*.

One solution to tackle medicine trafficking is educating patients and physicians on its impact on health and economic development.

*“Our achievement includes conducting 187 training classes for over 14,431 people on preventing health-oriented product smuggling, which increased awareness.” (Webda).*

*“One solution to combat medicine smuggling is to promote domestic health products and support national production.” (Mehr News Agency).*

Another solution is to eliminate smuggled medicines and their effect on reducing the motivation for smuggling.

*” Setting fire to the smuggled medicines in front of the smugglers has a powerful psychological impact.” (Mehr News Agency).*

Another report of media news declared that:

*“Creating a culture against drug trafficking is one of the ways to fight against medicine smuggling.” (Khabaronline)*.

## Discussion

As discussed in the literature review, the large size of the pharmaceutical market makes it an attractive target for exploitation, with greed being a common driving force behind corruption such as the smuggling of pharmaceuticals. However, several other factors contribute to the vulnerability of the pharmaceutical sector to the illegal act of medicine smuggling.

Our study used a content analysis approach of Iran’s news media to investigate the complex issue of medicine smuggling in Iran. We identified the key drivers that make the pharmaceutical sector of Iran susceptible to this illicit trade and proposed a comprehensive set of strategies to effectively address these vulnerabilities.

Our study concluded that various context-sensitive shortcomings related to the economic environment, governance, pharmaceutical information technology systems, and socio-cultural factors make the pharmaceutical sector vulnerable to smuggling. These shortcomings emphasize the multifaceted nature of medicine smuggling, which requires a multi-strategy approach to mitigate it.

The illegal transportation of pharmaceuticals in reverse direction creates obstacles for the patients to access necessary medicines at an affordable price. Our study reveals that economic sanctions, as well as internal economic issues within and outside the pharmaceutical industry of Iran, such as fluctuating exchange rates, misallocation of foreign exchange to importers and manufacturers instead of patients, and a defective pharmaceutical pricing mechanism, have hindered the growth of the industry. These results are consistent with previous studies conducted in Iran. A recent study by Ashrafi Shahmirzadi et al. concluded that various factors can affect the pricing mechanism in the pharmaceutical sector. However, the presence of conflicts of interest, lack of transparency, and absence of a robust framework have resulted in a situation where pharmaceutical pricing in Iran is determined arbitrarily [52]. According to Bastani et al., sanctions on pharmaceutical procurement in Iran have caused exchange rate fluctuations, restricted interaction with international organizations, and increased reverse smuggling [53]. Esfandiari et al. conducted a study that found that the public pharmaceutical sector of Iran is plagued with numerous transparency issues including conflicts of interest, centralization, monopoly, illegal payments, gifts, bribes, hidden power, and hoarding. These factors make the sector highly vulnerable to corruption [54]. A study by Fatemi Moghadam revealed that several factors contribute to the problem of medicine trafficking in Iran. These include the inflation rate, significant price differences between the black market and formal market, government policies, mismanagement of consumer pharmaceutical needs, and high prices of pharmaceuticals. Other factors include outdated laws, the expansion of trafficking networks, economic sanctions, inadequate domestic production, and inequitable drug distribution in the country. All of these factors have led to a failure to reduce medicine smuggling in Iran [25]. According to Garuba et al., Nigeria’s pharmaceutical system is at a higher risk of corruption due to the absence of clear guidelines to address conflicts of interest and anti-corruption measures. In addition, inappropriate policies and insufficient monitoring and evaluation are also significant contributing factors [18]. Kim and Tajima’s research supports our findings that penalties alone are not an effective deterrent in combating drug trafficking. Hosseini and colleagues have also verified that effective monitoring, cooperation, and educational programs at different levels can help lower the risk of counterfeit drugs and drug trafficking [22]. A recent study has highlighted that the illegal trade of drugs poses a major public health concern in Africa. While repressive actions are required to decrease the sale of fake drugs in the black market, they are not adequate on their own. It is crucial to provide advertising messages, raise awareness, and share information about drug trafficking with target groups to combat this issue effectively [55].

In economies that are controlled by the state, when there are too many market tensions, it ultimately leads to measures such as price controls, restrictions, and rationing. These bureaucratic measures are aimed at correcting market deviations, but they end up affecting both producers and consumers. In due course, producers start leaving the formal markets due to frozen and unrealistic prices, while pharmacies face difficulties keeping up with the rising demand. This results in consumers and patients turning to informal distribution channels. At this stage, the economy becomes susceptible to the black market.

The informal pharmaceutical market thrives due to its easy accessibility, which poses a challenge to both the healthcare and economic sectors. This is according to Dagrou and Chimhutu’s study, which is consistent with our findings [56]. To combat illicit pharmaceuticals, a comprehensive and multi-dimensional approach is needed to strengthen the regulatory framework and leverage inter-institutional collaboration and communication efforts to formulate an interagency strategy [3].

The study by Vande Walle and Ponsaers highlights the inequality between the informal and formal economies [57]. The high prices of products in the informal market exacerbate black markets, which are exploited by the formal market.

The DAROOYAR plan is an initiative by the Iranian government to tackle the issue of medicine trafficking. The plan involves readdressing the exchange and subsidy policies in the pharmaceutical sector to ensure that government subsidies allocated to medicines reach the end consumers. The ultimate goal of this plan is to reduce the instances of illegal medicine trafficking. This strategy is implemented to reform the pharmaceutical exchange rate policies to release pharmaceutical prices [2], and consequently reduce medicine trafficking. In this plan, the preferential exchange rate is transferred to consumers through increased prices by health insurance.

An intriguing discovery is that information technology systems can effectively reduce smuggling when developed in the best possible way and integrated. Additionally, a study conducted by Mackey and Cuomo has demonstrated that digital technologies can enhance transparency, accountability, and anti-corruption measures in medicines procurement, which brings added value to pharmaceutical supply chain track and trace policies [58].

The study shows that intersectoral collaboration within and outside the pharmaceutical industry is a major challenge. This negatively affects communication channels among stakeholders, making it harder to access reliable, safe, and integrated pharmaceutical information technology systems that can trace and track medicines throughout the supply chain. The study also found that public awareness of the risks associated with illicit pharmaceuticals, irrational prescriptions, and consumption of pharmaceuticals is poor. This could be due to unclear roles, responsibilities, and accountability among the numerous stakeholders [59] involved in the pharmaceutical sector. To combat the illicit pharmaceutical trade, it is recommended that the government builds upon existing inter-institutional collaboration and communication efforts to develop an interagency approach.

Strengthening electronic infrastructures has been identified as an effective way to combat drug trafficking, as several news sources have pointed out. Gaudiano et al. have confirmed this finding and emphasized that purchasing drugs online or through illegal channels is often seen as a cost-saving measure, even though it is illegal and poses significant risks. It is crucial to raise awareness about the dangers of buying drugs outside the legal system and without a prescription [14].

Medicine smuggling is significantly influenced by socio-cultural factors. Public awareness has a direct impact on the demand for smuggled medicines, which can lead to individuals being reluctant to report such activities, complicating law enforcement’s efforts. To combat this illicit trade, it is essential to increase public awareness about the consequences of medicine smuggling. When the general public is informed, they can make more informed choices and become vigilant against the dangers associated with smuggled medicines. Improving health literacy and raising public awareness about proper and rational pharmaceutical consumption patterns, as well as promoting a culture of not using smuggled medicines, can play a significant role in changing public health behavior and preventing medicine trafficking. Many studies have shown that this phenomenon can be reduced by appropriately and comprehensively promoting and informing people about how to use the pharmaceutical information system, as well as increasing their awareness of the side effects of smuggling drugs. Alizadeh Sameh and Pour Qahramani’s study also supports the result that increasing awareness about medicine trafficking is of the utmost importance, and one of the effective strategies to combat this phenomenon is to increase the public’s awareness. Additionally, a study conducted in Africa demonstrated that public awareness through television and radio announcements effectively increases public knowledge about the dangers of counterfeit drugs and trafficking, reducing purchases from the illegal market [60].

The government, in collaboration with civil society and the private sector, should establish public awareness campaigns aimed at providing consumers with comprehensive information regarding the health and economic consequences associated with medicine smuggling. Culturally tailored information initiatives should also be created. These campaigns should focus on at least four major areas of awareness. Firstly, they should address the health risks of counterfeit or substandard medicines, and how smuggled medicines can negatively affect the accessibility of affordable necessary medicines. Secondly, they should highlight the legal consequences of engaging in medicine smuggling. Individuals caught participating in these activities can face severe penalties, including imprisonment and fines. This would help increase the deterrence of penalties. Thirdly, the public should be encouraged to report suspicious pharmaceutical activities or medicines. Establishing an accessible reporting mechanism can help authorities identify and dismantle smuggling networks more effectively. Lastly, the campaigns should address the common perception among the public that imported foreign medicine is of higher quality compared to generic medicines. This perception is often linked to branding, marketing, and cultural biases. The belief that foreign medicines are superior can lead individuals to seek them out, even if they are smuggled or counterfeit. The campaigns should stress that the quality of a medicine is not solely determined by its origin, but by the regulatory processes in place to ensure its safety and efficacy. Many generic medicines are of high quality and meet the same safety and efficacy standards as their brand-name counterparts. It should also be highlighted that generic medicines are often more cost-effective than brand medicines [61], which can make them a more accessible and affordable option for patients. This affordability can have a positive impact on public health and healthcare costs. Our findings are in agreement with Alshakka et al.’s research, which showed that medicine smuggling in Yemen is a critical issue that has roots in the lack of public awareness of the dangers of smuggled drugs, and the lack of strict laws that penalize smugglers. There is also a lack of political will, effective policy, and laws and regulations [62].

Pharmacists play a crucial role in combating medicine smuggling due to their frontline position. Their perceptions, awareness, and practices are essential [63]. According to Gutorova et al., illegal internet pharmacies sell falsified, smuggled, and low-quality medicines, as well as prescription drugs without proper authorization [64].

The shortage of a supervisory workforce is a significant challenge in mitigating smuggling, including medicine smuggling in Iran. This shortage can have at least several negative consequences. First, A shortage of supervisory personnel can result in limited resources for conducting inspections and oversight of various sectors, including pharmaceutical distribution channels. Second, borders and ports are common entry and exit points for smuggled medicines. A shortage of supervisory staff can lead to less effective monitoring at these critical locations. Investigative efforts may be hampered, leading to difficulties in tracing the origins of smuggled medicines and apprehending those responsible. Law enforcement agencies may struggle to acquire and maintain the necessary technology, equipment, and training programs needed to combat smuggling effectively. With fewer supervisory personnel, there may be increased vulnerability to corruption and bribery within law enforcement agencies.

Poor intersectoral collaboration among stakeholders can hinder efforts to combat medicine smuggling in Iran. When different sectors and organizations do not effectively work together, it can lead to inefficiencies, duplication of efforts, and gaps in addressing the problem. The absence of strong leadership in the Ministry of Health with enough authority and poor political will can result in a lack of direction and accountability in collaborative efforts.

As stated in the literature review, an examination of the political-economic aspects surrounding illicit tobacco in Southern Africa and counterfeit medicines in Central America uncovers a disconcerting trend that political figures and government institutions are complicit in facilitating, endorsing, and safeguarding unlawful trade, even at the highest levels of governance. It is essential to recognize that, although there are distinct differences depending on the specific goods and circumstances, the perpetuation of black markets and illicit trade within developing economies is often exacerbated by profound and systemic governance failures and a lack of political resolve, rather than mere technical challenges that can be easily resolved. The presence of conflicts of interest within Iran’s pharmaceutical sector, as highlighted in prior research [54], renders the sector vulnerable to corrupt practices which should be declared and used in place regulations to be managed and avoided.

### Study limitations and strength

Our study has some limitations that need to be addressed. The first limitation is that our research relies only on media sources, which may limit the generalizability of the results to the pharmaceutical industry of Iran. Although we used a maximum variation sampling technique to enhance the data source triangulation, further research is needed to triangulate methods using other sources. The second limitation is that we only reviewed Persian news media, which means that non-Persian language media were excluded. This limitation may have impacted the scope of our findings. Additionally, we restricted the concept of smuggling to medicine trafficking, while smuggling is an expanded phenomenon that is prevalent in other necessary goods. This narrow focus may have undervalued the common factors that are considered as roots of smuggling in Iran. The issue of smuggling cannot be solved by the health sector alone. More studies are necessary to understand how to control this phenomenon from a broader perspective. Additionally, verifying the accuracy of news content is challenging due to the presence of partisan reporting. To tackle this issue, it is recommended to use a variety of data sources and methods in future research to verify the results.

This study marks one of the first attempts to analyze the various aspects of medicine smuggling in Iran by using specialized and general online news media. The study covers a significant period of six years, during which crucial politico-economic transformations such as economic sanctions, exchange rate fluctuations, and pharmaceutical exchange/subsidy reform took place. The analysis of media news during this extended period provides valuable insights into the issue of medicine smuggling in Iran.

## Conclusion

Our study examined the factors responsible for medicine smuggling in Iran and the strategies to combat it. The results showed that medicine smuggling is a multi-faceted issue affected by supply and demand sides’ factors including economic environment, governance and stewardship, information technology systems, and sociocultural factors. Therefore, a multi-strategy approach to combating the medicine smuggling is necessary. We highly recommend that public and health policymakers work collaboratively to develop clear strategies, improve regulations and supervision, enhance economic conditions in the pharmaceutical sector, strengthen governance and leadership, and conduct awareness campaigns to address the demand side of smuggling. According to the theme obtained from media analysis, some practical measures can be suggested to reduce this problem, such as strengthening operational training based on virtual networks, regulatory online shops, single currency price, strengthening and reforming the pharmaceutical plan, Cultivation, and education about drugs, drug use and trafficking, revision and updating of laws.

### Electronic supplementary material

Below is the link to the electronic supplementary material.

Supplementary Material 1

## Data Availability

The datasets analyzed during the current study are available in the website addresses of the studied news agencies. Moreover, the datasets analyzed during the current study are available from the corresponding author upon reasonable request.

## Abbreviations

WHO: World Health Organization
IRNA: Islamic Republic News Agency
ISNA: Iranian Students News Agency
IFDA: IIran Food and Drug Administration
IFDANA: Irans’ Food and Drug Administration News Agency
TTAC system: Tracing, Tracking, and Authentication Control
TPOI: Trade Promotion Organization of Iran
