# Supplementary material for: Drivers of vulnerability to medicine smuggling and combat strategies: a qualitative study based on online news media analysis in Iran
Source: BMC Health Serv Res. 2024 Mar 27;24:383. doi: 10.1186/s12913-024-10805-7 (PMC10976728; doi:10.1186/s12913-024-10805-7)
Supplement: Supplementary file 1 — Supplementary Material 1 [file 12913_2024_10805_MOESM1_ESM.docx]

**Supplementary file 1:** A brief general characteristics of the news sources included in the media analysis

**Table S1:** A brief general characteristics of the news sources and the number of news headlines

| **News Coverage** | **Name** | **Description** | **Medicine trafficking headlines** | **Website address** |
| --- | --- | --- | --- | --- |
| **General news platform** | Mehr News Agency | An Iranian news agency belonging to the Islamic Development Organization. It publishes news in six languages: Persian, English, Kurdish, Arabic, Urdu, and Istanbul Turkish. | 56 | https://www.mehrnews.com/ |
|  | Khabaronline | Part of the Khabar Media Group, including sections of Khabar-e-Junub, Khabar-e-Varzeshi, the weekly Siyahat & Tejarat, and the weekly literary Negah-e-Panjshanbeh. Other publications of this group are also considered prominent and sometimes experienced publications in their field. | 39 | https://www.khabaronline.ir |
|  | Iranian Students’ News Agency (ISNA) | The first news agency in Iran was established on the Internet for free in 2000, and access to its news is open to all audiences. | 44 | https://www.isna.ir/ |
|  | Islamic Consultative Assembly News Agency (ICANA) | A parliament news agency in Iran, established in 1998. Its purpose covers various political, social, economic, and international societal issues. | 19 | https://www.icana.ir/Fa |
|  | Islamic Republic News Agency (IRNA) | The official news agency of Iran. It publishes news about the university, social, economic, political, international, and sports issues. | 9 | https://plus.irna.ir |
|  | Donyay-e-Eghtesad Newspaper | An Iranian economic newspaper launched in 2002 and is currently the third most popular print newspaper in Iran, focusing on economic and social news. | 31 | https://donya-e-eqtesad.com |
|  | [Hamshahri Online](https://www.hamshahrionline.ir/) | A morning newspaper published in Iran. It has several active sections and covers the selected news from other online, visual, audio, and written media. | 24 | https://www.hamshahrionline.ir/ |
| Specialized in medical/health news | Sepid Online | Sepid Online is the first medical newspaper published weekly and posted the latest medical news and is managed by the Iran Medical Council. | 29 | http://sepidonline.ir/ |
|  | Webda | News and information site of the Ministry of Health and Medical Education (MoHME); It publishes information in health, medical services, and medical education. | 8 | https://behdasht.gov.ir/ |
|  | IFDANA | The Food and Drug Administration of Iran news site. It provides information about food and medicine, beverages, cosmetics, biology, supplements, and products. | 18 | https://ifdana.fda.gov.ir/fa/ |
| **Total** | | | **277** | |
